# Beyond molecular classification: integrating signaling, subcellular, and immune reprogramming in endometrial cancer

**DOI:** 10.3389/fonc.2026.1906951

**Published:** 2026-09-25

**Authors:** Miłosz Pietrus, Marcin Waligóra, Maciej W. Socha, Klaudia Skrzypek, Monika Kabzinska-Turek, Rene Laky, Iwona Gawron

**Affiliations:** 1 Department of Gynecology and Oncology, Faculty of Medicine, Jagiellonian University Medical College, Kraków, Poland; 2 Pulmonary Circulation Centre, Department of Cardiac and Vascular Diseases, Faculty of Medicine, Jagiellonian University Medical College, John Paul II Hospital in Kraków, Kraków, Poland; 3 Center for Innovative Medical Education, Department of Medical Education, Faculty of Medicine, Jagiellonian University Medical College, Kraków, Poland; 4 Department of Perinatology, Gynecology and Gynecologic Oncology, Faculty of Health Sciences, Collegium Medicum in Bydgoszcz, Nicolaus Copernicus University, Bydgoszcz, Poland; 5 Department of Transplantation, Institute of Pediatrics, Faculty of Medicine, Jagiellonian University Medical College, Kraków, Poland; 6 Department of Obstetrics & Gynecology, Medical University of Graz, Graz, Austria; 7 Chair of Gynecology and Obstetrics, Faculty of Medicine, Jagiellonian University Medical College, Kraków, Poland

**Keywords:** digital pathology, endometrial cancer, immunotherapy, mTOR signaling, NOTCH signaling, precision oncology, spatial transcriptomics, WNT signaling

## Abstract

Endometrial cancer (EC) is the most common gynecological malignancy in developed countries, with rising incidence rates that have not been matched by equivalent improvements in survival outcomes, particularly for high-grade and advanced-stage disease. The landmark molecular profiling performed by The Cancer Genome Atlas consortium transformed EC classification by identifying four reproducible subtypes with distinct prognostic profiles: POLE-ultramutated, mismatch repair-deficient, copy-number low, and copy-number high. This reclassification has directly informed clinical guidelines, and, most recently, enabled three practice-changing regulatory approvals that established chemoimmunotherapy as a new first-line treatment paradigm for advanced disease, although regulatory indications differ according to MMR status. Yet molecular heterogeneity within EC involves much more than just genomic alterations to encompass coordinated changes in developmental signaling pathways, subcellular protein redistribution, epigenetic regulation, and tumor–immune crosstalk. This narrative review synthesizes current evidence across several interconnected dimensions of EC biology: aberrant WNT/β-catenin and mTOR signaling with compartment-specific expression patterns; cancer stem cell markers, including CD133, SOX2, and OCT4; γ-glutamyl transferase as a redox-linked biomarker; NOTCH pathway dysregulation, whose context-dependent, occasionally paradoxical relationship with prognosis complicates direct translation of NOTCH-targeted therapies from other tumor types; an expanded landscape of tissue and circulating microRNAs for grade discrimination and prognosis, extending well beyond our own previously reported miR-375/miR-190b panel; and the prognostic and therapeutic relevance of tumor-infiltrating lymphocytes, together with T-cell receptor repertoire profiling as an emerging complementary immune biomarker. We also discuss emerging frontiers, including circulating tumor DNA as a minimal residual disease tool, single-cell and spatial transcriptomics revealing tumor microenvironment architecture, and artificial intelligence-augmented digital pathology. Building on these axes, we propose an integrated conceptual model in which developmental signaling, subcellular protein trafficking, epigenetic and stemness reprogramming, and immune remodeling are mechanistically coupled, rather than independent processes. A multidimensional, integrative framework incorporating these axes is essential for bridging the current gap between biological complexity and clinical precision in EC.

## 1 Introduction

Endometrial cancer (EC) remains the most frequently diagnosed gynecological malignancy in high-income countries, and its global incidence continues to rise in parallel with the increasing prevalence of obesity, metabolic syndrome, and an aging population (1, 2). In the US and Europe, endometrioid adenocarcinoma (EAC) accounts for approximately 80% of all cases (3). Although most EC patients present with early-stage disease and achieve favorable outcomes postoperatively, a clinically significant subset, particularly those with high-grade or advanced-stage tumors, carries a substantially worse prognosis, with five-year disease-specific survival rates as low as 17%–27% for stage IV disease (4), consistent with contemporary SEER-based five-year relative survival estimates ranging from approximately 95% for localized (stage I) disease to around 18% for distant/metastatic (stage IV) disease (5). Reliably predicting recurrence risk and individualizing therapeutic strategies beyond established clinicopathological factors remains a central challenge.

The traditional binary classification of EC into type I (estrogen-driven, low-grade endometrioid carcinoma) and II (non-estrogen-driven, high-grade non-endometrioid carcinoma) provided a useful but oversimplified framework (6). Comprehensive genomic profiling by The Cancer Genome Atlas (TCGA) consortium in 2013 represented a paradigm shift in EC classification, identifying four molecularly distinct subtypes: POLE-ultramutated tumors with an exceptionally favorable prognosis, mismatch repair-deficient (MMRd) tumors associated with microsatellite instability and responsiveness to immune checkpoint inhibition, copy-number low (endometrioid) tumors with intermediate prognosis, and copy-number high (serous-like) tumors carrying the worst prognosis (7). The subsequent development of the Proactive Molecular Risk Classifier for Endometrial Cancer (ProMisE) algorithm in 2017 enabled practical implementation of this molecular classification using standard immunohistochemical and molecular pathology techniques, facilitating translation into routine clinical practice (8). Subsequently, the European Society of Gynaecological Oncology/European Society for Radiotherapy and Oncology/European Society of Pathology (ESGO/ESTRO/ESP) 2020 guidelines formally incorporated molecular classification into risk stratification and adjuvant therapy selection (9). The therapeutic impact has been transformative; regulatory approvals for dostarlimab (RUBY trial) (10), pembrolizumab (NRG-GY018) (11), and durvalumab (DUO-E) (12), each in combination with carboplatin/paclitaxel, have established chemoimmunotherapy as a new first-line treatment paradigm for advanced EC, although regulatory indications differ according to MMR status. The integration of molecular features into the revised 2023 FIGO staging system reflects the degree to which genomic information has permeated EC clinical management (**Table 1**).

**Table 1:** Key milestones in the historical evolution of EC classification and clinical management.

Year	Milestone	Significance for EC	Ref.
1973	FIGO histological grading system established	Provided the first standardized histomorphological framework for EC risk stratification based on percentage of solid non-squamous growth; remains the foundation of clinical grading to date	(4)
1983	Bokhman’s binary classification: type I vs. II	Established the distinction between estrogen-dependent low-grade endometrioid carcinoma (type I) and non-estrogen-dependent high-grade disease (type II), forming the conceptual basis for histological subtyping for three decades	(6)
2000s	Recognition of significant interobserver variability in high-grade EC grading	Diagnostic consensus achieved in fewer than two-thirds of high-grade EC cases, motivating the search for objective molecular markers	(29, 30)
2013	(TCGA) Integrated genomic, transcriptomic and proteomic characterization of endometrial carcinoma	Identified four molecularly distinct subtypes: POLE-ultramutated, MMRd, copy-number low, and copy-number high. Each has a distinct prognostic profile; a paradigm shift from morphological to molecular classification	(7)
2017	ProMisE classifier	Translated TCGA subtyping into a clinically applicable four-class classifier using IHC and PCR, enabling routine pathological implementation	(8, 33)
2020	ESGO/ESTRO/ESP guidelines integrate molecular classification	First international guidelines to incorporate molecular subtype into risk stratification and adjuvant therapy algorithms; MMRd tumors recognized for immunotherapy candidacy	(9)
2022	KEYNOTE-775: lenvatinib plus pembrolizumab superior to chemotherapy in advanced EC	Established lenvatinib–pembrolizumab as a second-line standard of care, regardless of MMR status, cementing immunotherapy in EC management	(78)
2024	Chemoimmunotherapy established as a first-line standard for advanced EC	FDA approvals for dostarlimab (RUBY), pembrolizumab (NRG-GY018), and durvalumab (DUO-E), each combined with carboplatin/paclitaxel, transformed the first-line treatment landscape, although regulatory indications differ according to MMR status	(10–12)
2025	Revised 2023 FIGO staging system incorporates molecular features; next-generation sequencing-based classifiers validated as superior	Integration of POLE mutation and MMR status into staging; next-generation sequencing outperforms ProMisE in certain cohorts for copy-number classification	(32, 101)

Yet the four TCGA subtypes do not completely capture the biological heterogeneity of EC, particularly within the copy-number low and high groups. Growing evidence, including a body of work from our research group, suggests that EC is characterized by dynamic molecular reprogramming across multiple dimensions: signaling pathway dysregulation (13–15), subcellular protein redistribution (16–18), epigenetic regulation by small non-coding RNAs (19–22), metabolic reprogramming (15, 23, 24), and tumor–immune crosstalk (25–27). Their integrated examination within a single conceptual framework remains limited. Accordingly, the development of molecularly driven and biomarker-driven clinical trials is recommended to further advance precision medicine in the management of patients with endometrial carcinoma (28). This review synthesizes evidence across several interconnected axes of EC biology.

## 2 Literature search strategy

This is a narrative, rather than a systematic, review. Literature was identified through searches of PubMed/MEDLINE and Google Scholar using combinations of the terms “endometrial cancer,” “endometrial carcinoma,” “molecular classification,” “WNT signaling,” “mTOR,” “NOTCH,” “CD133,” “SOX2,” “OCT4,” “gamma-glutamyl transferase,” “microRNA,” “tumor-infiltrating lymphocytes,” “T-cell receptor repertoire,” “circulating tumor DNA,” “spatial transcriptomics,” and “digital pathology.” Foundational and mechanistic literature establishing core biological concepts still in use today (e.g., NOTCH/ATOH1 signaling, γ-glutamyl transferase redox biology, WNT ligand biology, and the original Bokhman type I/II classification) was considered without date restriction. Literature addressing clinical practice, molecular classification, and emerging biomarkers was restricted to 2013–2026, coinciding with the TCGA-era reclassification of EC that anchors this review. We prioritized landmark international publications (e.g., TCGA, ProMisE, ESGO/ESTRO/ESP guidelines, and phase III immunotherapy trials) and, where relevant, systematic reviews and meta-analyses over single-institution studies. In addition to this broader literature, we intentionally incorporated a series of studies previously published by us on subcellular protein localization [WNT-1, mechanistic target of rapamycin (mTOR), and CD133], γ-glutamyl transferase, and miRNA grading signatures in EC, as these represent complementary investigations of signaling, subcellular, and epigenetic dimensions of the same condition. The biomarkers and pathways examined in our own studies were themselves originally selected on the basis of prior literature and established biological relevance rather than arbitrarily; throughout this review, our findings are presented alongside, and where possible directly compared with, independent studies addressing the same markers or pathways to provide a balanced representation of the evidence.

## 3 The molecular classification paradigm: from histology to genomics

Before the molecular era, EC grading was exclusively determined by histomorphological criteria, most prominently the percentage of solid non-squamous, non-morular growth assessed by hematoxylin and eosin (H&E) staining. The FIGO grading system assigns grade 1 (G1) to tumors with less than 5% solid growth, grade 2 (G2) to those with 6%–50%, and grade 3 (G3) to those exceeding 50% (4). Despite widespread clinical application, this system is subject to significant interobserver variability, with diagnostic consensus achieved in fewer than two-thirds of high-grade EC cases (29, 30). These limitations underscore the need for objective, reproducible molecular markers.

The integrated genomic, transcriptomic, and proteomic characterization of EC provided the molecular foundation for this transition. TCGA profiling identified POLE exonuclease domain mutations, conferring an ultramutator phenotype with excellent prognosis; MMR deficiency, attributable to somatic MLH1 promoter hypermethylation in most sporadic cases or Lynch syndrome germline mutations, establishing a subgroup with sensitivity to checkpoint inhibition; and copy-number high tumors, characterized by TP53 mutations requiring escalated therapeutic approaches (7).

Clinically applicable classifiers, most notably ProMisE and the PORTEC molecular classification, enabled prospective integration of molecular subtyping into trial design and clinical guidelines (8, 31). Recent evidence indicates that next-generation sequencing-based classification outperforms surrogate immunohistochemistry (IHC)-based ProMisE in certain cohorts, particularly for copy-number subtype determination (32). A clinically applicable molecular-based classification using surrogate IHC markers continues to enable implementation outside specialist molecular pathology centers (33). International Society of Gynecological Pathologists consensus recommendations have further endorsed a multimodal classification scheme combining POLE mutational analysis with p53 and DNA mismatch repair IHC as a clinically pragmatic surrogate for complete TCGA characterization (34).

The impact of molecular classification on practice has been broad: POLE-mutated tumors now qualify for de-escalation of adjuvant radiotherapy in current guidelines (9). Its limitations, however, remain relevant. The four subtypes represent broad categories that do not capture the full heterogeneity within each group, and molecular classification at the DNA level does not comprehensively reflect the dynamic interplay among signaling pathways, protein trafficking, non-coding RNA regulation, and the immune microenvironment. Addressing these aspects requires a complementary and integrative approach to EC biology, as subsequently discussed.

## 4 Signaling pathway aberrations

### 4.1 WNT/β-catenin signaling

The WNT/β-catenin signaling pathway regulates cyclic endometrial proliferation and differentiation and is aberrantly activated in approximately 40% of EC cases, most commonly through CTNNB1 (encoding β-catenin) or APC mutations (13, 35, 36). WNT pathway dysregulation promotes tumor progression by sustaining cellular proliferation, inhibiting apoptosis, and maintaining cancer stem cell (CSC) properties (14). Clinically, CTNNB1 mutation and consequent WNT/β-catenin pathway activation have been directly linked to disease recurrence: an analysis of TCGA data confirmed that CTNNB1 mutations are enriched among recurrent low-risk EC cases, and functional studies in EC cell lines showed that direct inhibition of β-catenin transcriptional activity, rather than upstream WNT ligand secretion, selectively reduced viability in CTNNB1-mutant EC models, identifying β-catenin transcriptional output itself as a therapeutic vulnerability distinct from ligand-level WNT blockade (37).

Among WNT ligands, downregulation of WNT-7A expression is associated with improved overall survival in EC patients (38), while WNT-4, -2, -3, and -5A expression is downregulated in EC, suggesting that the pathway exerts complex, ligand-specific regulatory effects (39). WNT-10B expression correlates with histological type, FIGO stage, and lymphatic metastasis, indicating its involvement in disease progression (40). Notably, this ligand-level scenario is not internally consistent with a simple “WNT activation is oncogenic” model: if global WNT/β-catenin activation drives proliferation and CSC maintenance, the finding that WNT-7A loss, rather than gain, correlates with better survival implies that individual ligands can act divergently from, or even in opposition to, the overall transcriptional output of the pathway (14, 38). This mirrors the well-documented context-dependent duality described for NOTCH signaling (Section 7) and cautions against treating “WNT pathway activation” as a single, uniformly prognostic entity; ligand-, receptor-, and compartment-specific data, not just pathway-level activation status alone, are likely required to resolve these apparent contradictions.

WNT-1, one of the less extensively studied WNT ligands in EC, has emerged as a potentially informative compartment-specific biomarker (**Table 2**). In a cohort of 64 EC patients spanning all FIGO stages and grades (16), we found membranous WNT-1 expression to be negatively associated with higher tumor grade, with an area under the receiver operating characteristic curve (AUC) of 0.70 (P = 0.008; cut-off ≤ 5%, sensitivity 54.2%, specificity 90%) for excluding G3 tumors. Cytoplasmic WNT-1 showed a complementary positive association with high-grade disease (AUC 0.64, P = 0.038; cut-off >30%, sensitivity 100%, specificity 27.5%). Multivariable logistic regression confirmed both associations independently: for every 1% increase in cytoplasmic WNT-1-positive cells, the odds of G3 disease increased 274.9-fold (OR = 274.9, 95% CI 5.1–14,690.2, P = 0.006), while for every 1% increase in membranous WNT-1-positive cells, the odds of G3 disease decreased approximately 11-fold (OR = 0.09, 95% CI 0.009–0.89, P = 0.04). Direct pairwise comparison of the three ROC curves (membranous WNT-1, cytoplasmic WNT-1, nuclear mTOR) showed no statistically significant differences in discriminative performance (P > 0.26 for all), indicating comparable rather than superior individual predictive value.

**Table 2:** Summary of molecular biomarkers reviewed in this article, with methods, key findings, and clinical relevance.

Marker	Method	Key findings	Clinical relevance	Ref.
WNT-1 (membranous)	IHC	Negatively associated with tumor grade; AUC 0.70 (cut-off ≤5%; specificity 90%) for G3 exclusion	High specificity for ruling out high-grade disease; compartment-specific scoring required	(16)
WNT-1 (cytoplasmic)	IHC	Positively associated with high-grade tumor; AUC 0.64 (cut-off >30%, sensitivity 100%, specificity 27.5%)	May help identify high-grade disease; potentially most informative in combination with nuclear mTOR	(16)
mTOR (nuclear)	IHC	Positively associated with high-grade tumor and FIGO IB–IV; AUC 0.76 (sensitivity 70.8%, specificity 77.5%)	Highest AUC within the evaluated WNT-1/mTOR panel; however, pairwise ROC comparisons did not demonstrate statistically significant superiority over the other markers; predicts advanced FIGO stage	(16)
CD133 (nuclear)	IHC	Increases with tumor grade (G1 27.9% vs. G3 75.7% cells); positively associated with FIGO IB–IV and tumor angioinvasion; AUC 0.79 for G3	Identifies high-risk patients; associated with aggressive disease and angioinvasion	(17)
CD133 (membranous)	IHC	Decreases with grade; negatively correlated with angioinvasion; AUC 0.76 for G3 exclusion	Higher membranous expression associated with lower grade; potential favorable indicator	(17)
SOX2	IHC (FIGO stage I)	Positive in 89.9% specimens; higher expression in G3 vs. G2 (P = 0.020) and G1 (P = 0.008); SOX2/OCT4 co-expression restricted to G1 (R = 0.51, P = 0.031); age positively correlated with SOX2 (P = 0.003)	Reflects tumor dedifferentiation in stage I EC; limited independent prognostic value due to correlation with grade	(52)
OCT4	IHC (FIGO stage I)	Positive in 66.7% specimens; no grade-dependent variation; co-expression with SOX2 only in G1 (R = 0.51, P = 0.031); elevated in hypertensive patients	Does not independently reflect tumor grade in early EC; non-interchangeable oncogenic role vs. SOX2	(52)
GGT (apical membrane)	IHC	Positive in 92.9% (52/56) of G1–2 EAC and 91.7% (55/60) of controls vs. 23.8% (10/42) of G3 (P < 0.001); median score 2.0 in normal/G1–2 vs. 0.0 in G3 (P < 0.001); multivariate regression: independently associated with grade (P < 0.001) and FIGO stage (P = 0.002)	Loss of apical GGT is associated with high-grade EC; potential risk stratification biomarker in postmenopausal patients with EAC	(18)
miR-375	NGS + qPCR	Most significant discriminator between G3 and G2 EC; validated in the TCGA cohort; targets PDK1, JAK2, IGF1R, AEG-1	Candidate molecular grading adjunct; superior to histomorphology for G3 vs. G2 discrimination	(20, 70)
miR-190b	NGS + qPCR	Downregulated in G3 vs. G2; validated in the TCGA cohort; robust across clinical covariates	Independent predictor of high-grade EC; potential molecular grading panel element	(70, 72, 73)
NOTCH1–4	qPCR + Western blot	Downregulated in stage I EAC vs. adjacent non-tumor tissue (1.6- to 5-fold reduction). Higher reduction in later stages.	Findings consistent with a possible tumor-suppressive role during early transformation, with context-dependent prognostic effects in established tumors; potential biomarkers for tumor differentiation stratification	(64)
NOTCH2, NOTCH3, NOTCH4, JAG2, HES1 (intratumoral)	qPCR	Higher intratumoral expression independently associated with worse overall and disease-free survival in uterine cancer	Prognostic stratification within EC; identifies higher-risk tumors despite overall pathway attenuation vs. normal tissue	(65)
Tissue miRNA panel (miR-205, miR-200 family, miR-135b/-182/-183/-223)	Systematic review (26 studies, n = 1,400)	Six-miRNA panel achieved AUC 0.961, sensitivity 91%, specificity 94% for EC detection	Independent, multi-study support for miRNA-based diagnosis beyond our own G2/G3 panel	(21)
Circulating miRNA panels	Systematic review (33 individual miRNAs, six panels)	miR-200b/-200c/-203/-449a panel associated with myometrial invasion; seven individual miRNAs linked to grade, stage, and survival	Broadens circulating-biomarker evidence base beyond single-institution studies	(22)

NGS, next-generation sequencing; qPCR, quantitative polymerase chain reaction.

The intersection of mTOR signaling is mechanistically relevant: mTOR activates WNT/β-catenin transcriptional outputs (41), while mTORC1 suppresses WNT/β-catenin through Dishevelled-dependent regulation of Frizzled receptor levels (42), a dual relationship that, if operative in EC, could plausibly contribute to a self-reinforcing oncogenic circuit in high-grade disease. Therapeutic targeting presents both opportunities and challenges: mTOR inhibitors such as everolimus have demonstrated clinical activity in EC, but WNT blockade risks impairing tissue homeostasis (43). **Figure 1** illustrates the functional convergence of WNT and mTOR signaling, together with their downstream immune consequences.

**Figure 1:**
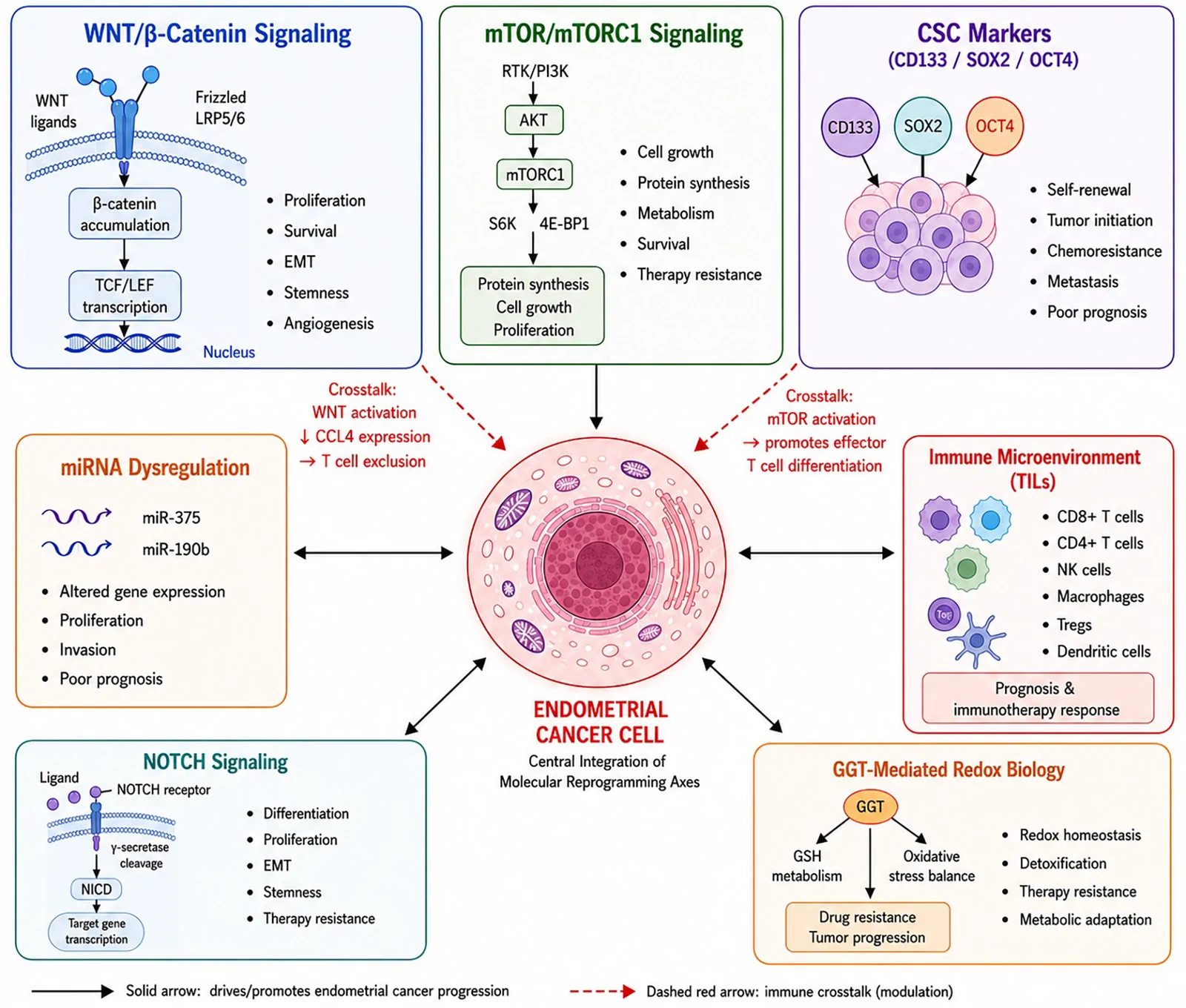
Integrated molecular and subcellular reprogramming in EC. Key axes of molecular reprogramming: WNT/β-catenin signaling, driving proliferation, survival, epithelial–mesenchymal transition (EMT), stemness, and angiogenesis; mTOR/mTORC1 signaling, promoting cell growth, protein synthesis, metabolism, survival, and therapy resistance; CSC markers associated with self-renewal, tumor initiation, chemoresistance, and metastasis; miRNA dysregulation, with miR-375 and -190b downregulation associated with tumor grade in EC, and broader mechanistic roles in proliferation, invasion, and poor prognosis primarily established in other cancer models; TILs determine prognosis and immunotherapy response; NOTCH signaling, regulating differentiation, EMT, and therapy resistance; and GGT-mediated redox biology, contributing to drug resistance and tumor progression. Solid arrows indicate pro-tumorigenic effects on EC progression. Dashed red arrows denote immune crosstalk: WNT activation suppresses CCL4 expression, promoting T cell exclusion, whereas mTOR activation promotes effector T cell differentiation. TCF, T cell factor; LEF, lymphoid enhancer factor; GSH, glutathione; NICD, notch intracellular domain; NK cells, natural killer cells.

Altogether, the WNT/β-catenin literature in EC remains largely correlative and derived from small, single-institution immunohistochemical cohorts (e.g., our own WNT-1 study, n = 64, which lacked a healthy control group and relied on a single validation method) (16) or retrospective TCGA re-analyses; ligand-specific functional mechanisms explaining divergent prognostic associations have not been directly tested in EC tissue or models, and prospective, multicenter validation of any compartment- or ligand-specific WNT biomarker remains lacking.

### 4.2 mTOR signaling

mTOR is a central integrator of growth factor signaling, nutrient availability, and metabolic status, operating through mTORC1 and mTORC2 complexes (15). In EC, mTOR pathway activation is among the most frequent molecular events, with PI3K/AKT/mTOR cascade alterations in up to 93% of endometrioid EC cases (7). The pathway promotes EC cell proliferation and metabolic reprogramming, and its dysregulation is implicated in resistance to hormonal therapy and cytotoxic agents (23, 24).

Beyond its canonical cytoplasmic role in regulating protein synthesis through S6K1 and 4E-BP1 phosphorylation, nuclear mTOR localization carries distinct functional significance in tumor cells, where it acts as a transcriptional integrator to regulate genes involved in cell growth, apoptosis, and mitochondrial oxidative metabolism (44). Nuclear mTOR expression is associated with disease progression in prostate cancer and multiple myeloma, where increased nuclear translocation correlates with poor prognosis (45, 46). In our previous study (16), we identified a positive association between nuclear mTOR expression and higher tumor grade and FIGO stage IB–IV, with an AUC of 0.76 (P < 0.001) at a cut-off of >10% (sensitivity 70.8%, specificity 77.5%), representing the strongest single predictor among the WNT-1/mTOR panel; on multivariable logistic regression, each 1% increase in nuclear mTOR-positive cells increased the odds of G3 disease 1,054.7-fold (95% CI 17.3–64,354.1, P = 0.0009). Nuclear mTOR expression was also significantly higher in FIGO stage IB–IV tumors than in stage IA disease (median 20% vs. 5%, P = 0.03). No individual marker was superior based on AUC comparisons, supporting a panel-based approach.

In addition to its diagnostic association with grade, mTOR activation mechanistically links proliferation to a broader stemness–metabolism axis. In PI3K/AKT/mTOR-driven CSC models, mTORC1 activity sustains self-renewal and tumor-initiating capacity while simultaneously licensing the anabolic metabolic program, including enhanced glycolytic flux, lipid biosynthesis, and adaptive responses to hypoxia via HIF-1α, essential to support that self-renewal, and confers relative resistance to apoptosis and conventional cytotoxic therapy in the CSC-like compartment (47). Although this mechanistic framework has been primarily established in other solid tumors (chiefly breast cancer), its core logic—that mTOR functions not merely as a proliferative signal but as a coordinator of the metabolic and survival programs underlying a stem-like, therapy-resistant cell state—is directly applicable to the grade-associated nuclear mTOR redistribution described earlier, and offers a plausible mechanistic explanation for why nuclear mTOR positivity tracks with the more aggressive, dedifferentiated end of the EC grading spectrum. This mechanistic association has not, however, been tested in EC-derived cells or tissue, and should thus be regarded as an extrapolated hypothesis rather than an EC-validated finding.

As with the WNT-1 findings above, the mTOR data presented here are derived from a single retrospective cohort (n = 64) at one institution (16). Notably, the original study included no healthy endometrial control group for comparison; all 64 patients had a cancer diagnosis, precluding direct assessment of these markers relative to normal endometrium, and validation relied on a single molecular method (IHC) without cross-validation by an independent technique or correlation with overall survival data. Causal, mechanistic confirmation of the proposed stemness–metabolism association in EC tissue is presently lacking.

## 5 CSCs and pluripotency-associated markers

### 5.1 CD133 as a compartment-specific biomarker

CD133, encoded by PROM1, is a transmembrane glycoprotein canonically localized to plasma membrane protrusions, and its expression has been linked to cancer-initiating potential, chemotherapy resistance, and aggressive tumor behavior across multiple solid tumors (48). Across the broader oncology literature, CD133 positivity has been reported as both a marker of poor prognosis and, in other series, as prognostically neutral or even favorable—a discrepancy long attributed to inconsistent antibody clones, glycosylation-dependent epitope masking, and, most relevantly here, failure to distinguish subcellular compartments within the same tumor (48). This inconsistency mirrors the same context-dependent pattern already discussed for NOTCH (Section 7) and WNT-7A (Section 4.1): a single biomarker whose net prognostic direction depends on precisely which molecular pool, i.e., membranous, cytoplasmic, or nuclear, is actually being measured, rather than on total protein abundance.

When nuclear, cytoplasmic, and membranous compartments are separately analyzed, a coherent picture emerges (17). We previously found that nuclear CD133 expression progressively increased from G1 to G3, while plasma membrane CD133 expression decreased with increasing grade. Nuclear CD133 was positively associated with FIGO stages IB–IV and tumor angioinvasion. The AUC for nuclear CD133 in predicting G3 disease was 0.79, and for angioinvasion was 0.75.

The mechanistic basis for nuclear CD133 accumulation likely involves cooperative transcriptional activity with STAT3. In hepatocellular carcinoma, IL-6/STAT3 signaling has been shown to directly upregulate CD133 transcription; STAT3 binds the CD133 promoter and cooperates with NF-κB and HIF-1α to sustain CD133 expression and a stem-like phenotype under hypoxic conditions (49). Notably, STAT3 itself is a transcription factor with established roles in endometrial carcinogenesis (50).

Beyond its role as a static biomarker, this compartmental switch plausibly contributes to the intratumoral heterogeneity that drives invasion, recurrence, and treatment resistance in EC. If nuclear CD133–STAT3 signaling marks a transcriptionally distinct, more stem-like subpopulation within a tumor, then grade-dependent increases in the nuclear-CD133-positive fraction would be expected to co-occur with, and potentially help generate, the phenotypically diverse, plastic cell populations increasingly documented by single-cell and spatial transcriptomic studies of EC (see Section 10.2). Under this model, nuclear CD133 positivity would identify a specific, targetable subpopulation disproportionately responsible for angioinvasion and, by extension, recurrence, a hypothesis that can be directly assessed with paired IHC and single-cell profiling of the same specimens. Such an investigation is yet to be conducted in EC. The broader phenomenon of membrane-to-nucleus translocation of transmembrane proteins has emerged as a key mechanism of cellular plasticity in malignancy and warrants further investigation in EC.

These findings should nonetheless be cautiously interpreted: they are derived from a single retrospective, single-institution cohort without external validation; the IL-6/STAT3-CD133 mechanism itself has only been demonstrated in hepatocellular carcinoma (49) rather than directly in EC tissue, and no functional (knockdown/overexpression) studies have yet established that nuclear CD133 is causally responsible for angioinvasion in EC rather than merely co-occurring with it.

### 5.2 SOX2 and OCT4: pluripotency factors in endometrial tumorigenesis

SOX2 and OCT4 are master regulators of pluripotency and self-renewal in embryonic stem cells, where they cooperatively activate a core transcriptional network sustaining stemness. Their aberrant expression in somatic cancers is linked to CSC maintenance, epithelial–mesenchymal transition, chemoresistance, and metastatic potential (51). SOX2 and OCT4 expression was characterized in early-stage EC using IHC in 69 stage I EAC specimens (52). SOX2 immunopositivity was detected in 62 of 69 specimens (89.9%), significantly more frequently than OCT4 positivity (46 of 69 specimens, 66.7%; P = 0.015). SOX2 expression was significantly higher in G3 tumors compared to both G2 (P = 0.020) and G1 (P = 0.008) tumors, while OCT4 expression showed no grade-dependent variation. A positive co-expression correlation between SOX2 and OCT4 was observed exclusively in G1 tumors (R = 0.51, P = 0.031), suggesting that their functional coordination is disrupted as tumors dedifferentiate. While SOX2 overexpression reflects tumor dedifferentiation, its utility as an independent prognostic biomarker at stage I is limited by its close correlation with histological grade. The selective predominance of SOX2 over OCT4 in high-grade disease parallels SOX2 elevation reported in other tumor types, including bladder cancer (53) and small cell lung cancer (54), suggesting that these transcription factors have non-interchangeable oncogenic roles in EC (**Table 2**).

These findings are limited by their derivation from a single retrospective cohort restricted to stage I EAC (n = 69) (52), which precludes assessment of SOX2/OCT4 behavior in advanced-stage or non-endometrioid disease; the cross-sectional design cannot establish whether the loss of SOX2/OCT4 coexpression coordination is a cause or consequence of dedifferentiation; and, as with WNT-1, mTOR, and CD133, no external validation cohort has been reported.

## 6 Redox biology and γ-glutamyl transferase in EC

GGT, a membrane-bound enzyme central to glutathione metabolism and intracellular redox homeostasis (55, 56), provides tumor cells with cysteine for glutathione resynthesis under oxidative stress conditions. In malignant tissues, GGT expression has been implicated in tumor progression through several non-mutually exclusive mechanisms: cysteine provision for glutathione resynthesis, reactive oxygen species generation through pro-oxidant reactions that contribute to genomic instability, and protection against apoptosis (57). Normal endometrium expresses GGT on the apical surface of glandular epithelial cells across the menstrual cycle (56).

The first detailed analysis of GGT expression in endometrioid EC tissue employed IHC in a cohort of 98 EC patients and 60 postmenopausal controls (18). Apical GGT staining was observed in 52 of 56 (92.9%) G1–2 EAC specimens and 55 of 60 (91.7%) controls, compared with only 10 of 42 (23.8%) G3 specimens (P < 0.001). Comparable high median apical GGT expression was confirmed in normal postmenopausal endometrium (median score 2.0, SEM 0.28) and G1–2 EAC (2.0, SEM 0.26), while G3 EAC specimens showed markedly reduced apical GGT expression (0.0, SEM 0.07; P < 0.001 vs. both comparators) (**Figure 2**). Following stratification by FIGO stage, the highest apical GGT expression was observed in stage IA tumors (4.0, SEM 0.47), comparable to controls, and the lowest in stage II disease (0.0, SEM 0.64); stage IB (1.0, SEM 0.24) and IIIA–C (1.0, SEM 0.16) tumors showed intermediate expression. Multivariate regression confirmed that GGT apical expression was independently associated with both tumor grade (P < 0.001) and FIGO stage (P = 0.002), whereas GGT cytoplasmic expression showed no relationship with grade, stage, age at diagnosis, parity, age at menarche, or BMI (18). A secondary finding, i.e., increased peritumoral stromal GGT positivity in G1–2 EAC (75%, 21/28) compared with controls (51.67%, 31/60; P = 0.038), with no stromal staining in G3, indicates a possible paracrine or field-effect component to GGT dysregulation that has not yet been mechanistically explored. These findings suggest that loss of apical GGT accompanies dedifferentiation in EC; however, in non-polarized neoplastic cells, redistribution of GGT to the entire cell surface may augment tumor invasiveness in ways not captured by the apical staining score.

**Figure 2:**
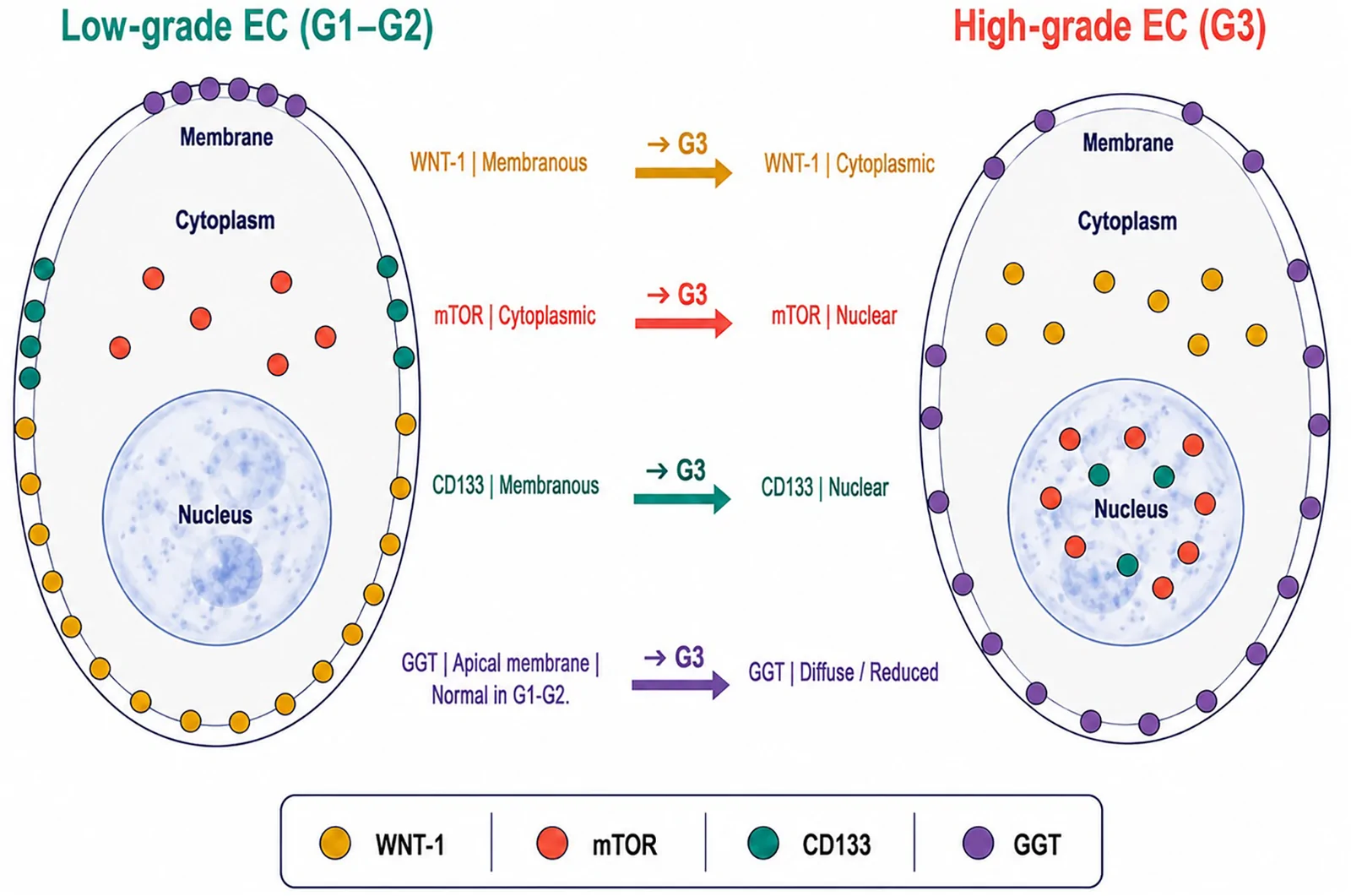
Subcellular localization schema for prognostically relevant proteins in EC. Schematic of an EC cell illustrating grade-dependent compartmental redistribution of WNT-1 (membranous in G1-2; cytoplasmic in G3), mTOR (cytoplasmic in G1-2; nuclear in G3), CD133 (membranous in G1-2; nuclear in G3), and GGT (apical membrane in G1-2; diffuse/reduced in G3).

These findings should be interpreted with several important caveats. First, they are derived from a single retrospective, single-institution cohort (98 EAC cases, 60 controls) without external replication (18); to our knowledge, no independent EC-specific GGT study has been published to confirm or refute this grade-associated apical loss. Second, the postmenopausal-only design leaves the relationship between GGT expression and grade/stage unexamined in premenopausal EC, which may differ given hormonal influences on the cycling endometrium. Third, no functional studies have established whether loss of apical GGT is mechanistically causal for EC dedifferentiation, an epiphenomenon of it, or a marker of another, unmeasured process; notably, correlation with grade does not itself establish direction of causality. Prospective studies measuring GGT tissue expression alongside serum GGT and oxidative stress markers in larger, multi-institutional cohorts are warranted to clarify its prognostic independence from established clinicopathological variables.

## 7 NOTCH pathway dysregulation in EC

The basic helix-loop-helix transcription factor ATOH1 is a master regulator of cellular differentiation across multiple lineages, with well-characterized roles in inner ear hair cell development and intestinal secretory cell specification (58, 59). The NOTCH signaling pathway canonically antagonizes ATOH1 by driving HES family transcriptional repressors that directly suppress ATOH1 promoter activity (60, 61), and γ-secretase inhibition of NOTCH leads to ATOH1 upregulation and cellular differentiation in colorectal cancer (62).

In EC, NOTCH pathway components show altered expression relative to normal endometrium and carry prognostic value (63). Lachej et al. demonstrated that mRNA levels of all four NOTCH receptors (NOTCH1–4), ligands (JAG1, JAG2, and DLL1), and the canonical target gene HES1 are downregulated in stage I EAC compared with adjacent non-tumor tissue, with progressively lower expression in stage IB versus IA disease (**Table 2**). In contrast, when comparisons are made within tumors, higher intratumoral expression of NOTCH2, NOTCH3, NOTCH4, JAG2, and HES1 is independently associated with worse overall and disease-free survival in uterine cancer patients (64, 65). Polychronidou et al. (66) employed IHC to report that NOTCH2 and NOTCH3 protein expression is associated with increased relapse and mortality in a 204-patient cohort. These observations, although contradictory, reflect different reference comparisons: relative to normal endometrium, NOTCH signaling is globally attenuated in EC, consistent with a tumor-suppressive role for the pathway during malignant transformation; upon transformation, however, tumors that retain or recover higher NOTCH activity behave more aggressively, suggesting a context-dependent switch in pathway function within established disease (64–66). Such dual, context-dependent roles for NOTCH, where NOTCH acts as an oncogene in some tissues and a tumor suppressor in others, are well documented across malignancies and likely reflect cell-type-, ligand-, and microenvironment-specific downstream effects (67). This pattern contrasts with the more straightforward antagonistic ATOH1–NOTCH relationship described in intestinal and colorectal models. Whether ATOH1 itself is dysregulated in human EC tissue has not been systematically characterized and remains an open question.

Taken together, we interpret these findings to suggest that γ-secretase inhibitors and other NOTCH pathway-directed strategies developed in other tumor types (62), where pathway activity is consistently oncogenic, may not be directly translatable to EC, where pathway expression appears reduced overall but residual activity associates with worse outcomes. Receptor-subtype-resolved studies and functional validation in EC models are needed before NOTCH-targeted therapy can be rationally positioned in this condition.

The NOTCH literature reviewed here also has some important limitations. The studies cited draw on heterogeneous methodologies (qPCR-based mRNA quantification vs. IHC-based protein detection) and different reference comparators (adjacent non-tumor tissue vs. across-grade comparisons within tumors), likely contributing to at least part of the apparent contradiction between global pathway attenuation and adverse intratumoral prognostic value, independent of any true biological context-dependence. Cohort sizes are modest (204 patients in the largest IHC series) (66) and single-institution; no NOTCH biomarker discussed here has undergone prospective, multicenter validation, and the causal relationship between NOTCH expression and EC aggressiveness, as opposed to a purely associative one, remains unclear.

## 8 miRNA-based transcriptomic alterations and tumor grade

### 8.1 Biological and diagnostic landscape of miRNAs in EC

miRNAs regulate gene expression post-transcriptionally and, considering their stability in formalin-fixed tissue and biological fluids, are attractive biomarker candidates for tissue and liquid biopsy applications (19, 20). A systematic review of 26 diagnostic studies encompassing 1,400 patients with EC identified 106 differentially expressed miRNAs across tissue, serum/plasma, and urine specimens, with miR-205, the miR-200 family (miR-200a/b/c, miR-141, miR-429), and miR-135b, -182, -183, and -223 showing frequent upregulation in EC relative to normal or benign tissue (21). Further, a six-miRNA panel (miR-205, -200a, -200c, -182, -183, -21) achieved an AUC of 0.961 (sensitivity 91%, specificity 94%) for distinguishing EC from hyperplasia or normal tissue in a validation cohort, though this and most of the underlying studies were small and methodologically heterogeneous (21, 68). A separate meta-analysis found miR-182 and -183 to be associated with poorer overall and recurrence-free survival, extending their relevance from diagnosis to prognosis (20, 69).

### 8.2 Tissue miRNAs associated with grade and prognosis

Beyond diagnostic panels, several tissue miRNAs track with tumor grade and differentiation status. We previously performed next-generation sequencing-based miRNA profiling of tumor specimens from 24 EC patients across G1–3, identifying differential expression patterns associated with tumor grade (70). Of 1,128 identified transcripts, only three distinguished G2 from G3: miR-375, miR-214-3p, and miR-190b. The comparatively small number of differentially expressed miRNAs between G2 and G3 reflects the well-recognized molecular continuum between these grades and the clinical difficulty of reliably distinguishing them using histomorphology alone (29). miR-375, the most significant discriminator between G2 and G3 disease, is a broadly expressed tumor suppressor targeting PDK1, JAK2, IGF1R, and AEG-1. Its expression is downregulated in multiple cancer types, including hepatocellular, esophageal, gastric, and head and neck cancers (71). In EC, reduced miR-375 expression is associated with positive lymph node status, reinforcing its role as a marker of aggressive disease. miR-190b, less extensively characterized, has been identified both as a tumor promoter in bladder carcinoma (70, 72) and as a tumor suppressor in lung cancer (73), illustrating the context-dependency of miRNA function that complicates mechanistic interpretation across the wider miRNA literature. Both miRNAs were validated in the TCGA cohort, and a random forest model built on the 10 most discriminative miRNAs achieved classification error rates of 10% (G1) and 14.3% each (G2, G3), substantially lower than the 37.5% morphological misclassification rate reported for high-grade EC (29, 70). These findings should be interpreted within the broader context of tissue–miRNA research. Independent work has implicated the miR-200 family in epithelial–mesenchymal transition via ZEB1/2 repression of E-cadherin and miR-205 in PTEN regulation (21, 74). Altogether, these findings suggest that multiple miRNA families, not just miR-375 and miR-190b, serve as candidate grading adjuncts.

### 8.3 Circulating miRNAs and liquid biopsy

A systematic review of miRNAs circulating in the serum or plasma of patients with EC highlighted 33 individual miRNAs and six multi-miRNA panels with significant expression differences between EC patients and healthy controls, including an miR-200b/-200c/-203/-449a panel associated with myometrial invasion and seven individual circulating miRNAs (miR-9, -21, -27a, -29b, -99a, -142-3p, -449a) associated with histological type, grade, FIGO stage, lymph node involvement, or survival (22). A broader meta-analysis across 11 studies reported that circulating miRNAs merit further evaluation as a prognostic factor in EC management but stopped short of recommending routine clinical use given study heterogeneity (20). Collectively, these data indicate that no single circulating miRNA yet outperforms multi-marker panels and that panels combining tissue and circulating miRNA data with molecular (TCGA) classification represent the most promising path toward clinical utility.

### 8.4 Limitations and translational challenges

Across both the tissue and circulating miRNA literature, several limitations recur. First, methodological heterogeneity due to the use of divergent profiling platforms (e.g., microarrays vs. RT-qPCR), reference/normalization genes, and specimen types (e.g., serum vs. plasma vs. urine) produces inconsistent, and sometimes directly conflicting, results for the same miRNA across studies (21, 22). Second, sample sizes are frequently small, and few studies have undergone external, multicenter validation. Third, most reported miRNAs are not EC-specific and show comparable dysregulation in other epithelial cancers, limiting standalone diagnostic specificity. Fourth, geographic representation is heavily skewed toward Asian and European cohorts, with essentially no data from Africa, South America, or Oceania (21). Finally, while our own G2/G3-discriminating panel (miR-375/-190b/-214-3p) performed well against histomorphology in TCGA validation cohort, similar to all EC miRNA panels published to date, it awaits prospective, multicenter validation before clinical implementation (70). At present, we view the miRNA literature as a complementary, still-maturing molecular grading adjunct to TCGA/ProMisE classification rather than a standalone diagnostic or prognostic tool.

## 9 Tumor-infiltrating lymphocytes and the immune microenvironment

### 9.1 TIL biology and prognostic significance

TILs encompass CD8+ cytotoxic T cells, CD4+ helper T cells, regulatory T cells (Tregs), and natural killer cells, with their relative abundance and spatial distribution reflecting the dynamic interplay between tumor immunogenicity and immune evasion (25). MMRd EC tumors elicit strong adaptive immune responses characterized by dense TIL infiltration, partly explaining their favorable prognosis and heightened sensitivity (75). High stromal TIL density is associated with improved disease-specific and overall survival in endometrioid EC, independent of stage and grade (76). A recent meta-analysis of 2,319 EC patients confirmed that high TIL levels, particularly CD8+ T cells, were significantly associated with better overall prognosis (HR = 0.484, 95% CI 0.411–0.556; P < 0.05) (77). The CD8+/Treg ratio is a particularly informative composite marker: Treg-dominant infiltrates promote immune escape and correlate with poorer outcomes, while high CD8+/Treg ratios reflect effective cytotoxic immunity.

### 9.2 Standardized TIL assessment and immunotherapy integration

The FDA approvals establishing combination chemoimmunotherapy as a first-line treatment paradigm for advanced EC [for dostarlimab (RUBY), pembrolizumab (NRG-GY018), and durvalumab (DUO-E), each combined with carboplatin/paclitaxel (10–12), with regulatory indications differing according to MMR status] have created an urgent need for reliable predictive biomarkers of response beyond MMR status. The prior establishment of lenvatinib–pembrolizumab as a second-line option (78) and these three front-line approvals together represent a transformation of the immunotherapy landscape in advanced EC. PD-L1 expression, microsatellite instability, and tumor mutational burden (TMB) each have predictive limitations in this context (79, 80). TIL density and composition may offer complementary or superior value in certain patient subgroups. Standardized TIL scoring methodologies, analogous to those developed for early breast cancer (81), are actively being developed for EC and may soon form part of the routine pathological report. A machine learning-based immune response signature derived from TCGA data has demonstrated independent prognostic value across EC molecular subtypes (82), supporting the clinical utility of quantitative immune cell assessment.

Several limitations temper these conclusions. TIL quantification methods vary considerably across the cited studies (manual counting vs. digital image analysis; stromal vs. intratumoral compartments), limiting direct comparability of reported hazard ratios and cut-offs, and no EC-specific TIL scoring system has yet been validated in the way it has been by Kos et al. for breast cancer (81). Much of the supportive evidence for machine learning-derived immune signatures comes from retrospective analysis of a single public dataset (82) rather than prospectively collected, independent cohorts; the CD8+/Treg ratio literature, drawn from smaller single-institution series, likewise awaits independent, multi-cohort validation. Prospective, biomarker-stratified trials directly comparing TIL-based stratification against MMR/TMB-based stratification in EC remain lacking.

### 9.3 Signaling–immune crosstalk

The mechanistic links between the signaling pathways discussed earlier and the immune microenvironment are increasingly recognized (**Figure 1**). WNT/β-catenin activation impairs cytotoxic T cell recruitment to tumors by suppressing CCL4-mediated dendritic cell infiltration, a mechanism sufficient in melanoma models to produce a T-cell-excluded phenotype independent of TMB (26). mTOR kinase differentially regulates effector and memory T cell lineage commitment, with mTORC1 promoting effector differentiation (27); because mTOR is simultaneously a tumor-intrinsic proliferative/stemness driver (Section 4.2) and a determinant of T-cell fate, tumor-cell-intrinsic mTOR activation and T-cell-intrinsic mTOR signaling within the same microenvironment may exert opposing net effects on antitumor immunity, a tension not yet resolved for EC specifically.

NOTCH signaling illustrates this tumor cell-to-immune cell crosstalk in more mechanistic detail (83, 84). In addition to its established role in T-cell development, tumor-cell NOTCH activation can reshape the composition, not merely the density, of the surrounding immune infiltrate: NOTCH ligand–receptor interactions between tumor and stromal/immune cells reportedly influence dendritic cell antigen-presenting capacity, skew macrophage polarization toward an immunosuppressive phenotype, and modulate regulatory T-cell recruitment and expansion, potentially favoring an immunosuppressive tumor–immune microenvironment over one dominated by effector cytotoxic populations. NOTCH-mediated effects on the tumor–immune microenvironment have additionally been associated with variable response to immune checkpoint blockade across tumor types, positioning NOTCH pathway status as a candidate contributor to the immunotherapy response heterogeneity observed within molecularly defined EC subgroups (see Section 12) (83, 84).

These interconnections suggest that the signaling and immune dimensions of EC are not parallel but functionally intersecting, such that tumor-intrinsic WNT/mTOR/NOTCH activation status may help explain why some tumors within the same molecular subtype (e.g., MMRd) respond to immunotherapy while others do not, and that composite immune-molecular signatures will likely outperform either dimension alone. It should be emphasized, however, that whether these same mechanisms (CCL4 suppression, mTORC1-dependent T-cell lineage commitment, and NOTCH-driven immune cell polarization) operate in the EC tumor–immune microenvironment remains currently unknown.

### 9.4 T-cell receptor repertoire analysis: a complementary immune readout

Beyond quantitative assessment of TIL density and composition, TCR repertoire profiling provides complementary information on T-cell clonality, diversity, and antigen-driven immune responses. Across solid tumors, a more clonally restricted intratumoral TCR repertoire is generally associated with improved survival, whereas greater diversity in peripheral blood tends to reflect broader systemic immune competence; high baseline intratumoral clonality has also been linked to improved response to anti-PD-1/PD-L1 checkpoint blockade in several cancer types (85, 86). In EC specifically, evidence remains limited: a recent integrated analysis of paired tumor and peripheral-blood TCR repertoires in molecularly characterized EC reported substantial immunological heterogeneity even within MMRd tumors, indicating that repertoire-based metrics may capture immune information not reflected by TMB or MMR status alone (87). Given this early-stage evidence base, TCR repertoire analysis should currently be regarded as an emerging, exploratory biomarker for prognosis and immunotherapy-response prediction in EC, requiring substantially larger and prospectively designed studies, alongside methodological standardization, a limitation affecting TCR profiling across cancer types generally, before clinical application (85).

## 10 Emerging frontiers: circulating tumor DNA, spatial omics, and artificial intelligence-augmented pathology

### 10.1 ctDNA and minimal residual disease

ctDNA has emerged as a promising non-invasive tool for risk stratification, prognosis, and early detection of recurrence, with a rapidly growing evidence base in EC (88, 89). Detecting pre-operative ctDNA mutations is associated with advanced stage, higher preoperative CA125, and subsequent disease recurrence; in a recent study, in 42% of patients with apparent clinical stage I disease, ctDNA detected stage II/III confirmed on final pathology (90). Post-operative ctDNA clearance preceded normalization of CA125 in serial monitoring studies, positioning ctDNA as a more sensitive early recurrence marker than conventional biomarkers. ctDNA mutations were identified 2–5 months prior to clinical or radiological progression in patients developing recurrence.

Post-surgical ctDNA-based MRD monitoring has shown high sensitivity for detecting occult microscopic residual disease when assessed within the first 4–8 weeks postoperatively (88). The Signatera MRD test has received broader regulatory coverage for pan-cancer immunotherapy monitoring, which may facilitate future evaluation of ctDNA-based monitoring in EC, although its specific validation in EC remains to be established (91). Pap-derived cell-free tumor DNA has recently demonstrated superior sensitivity to plasma ctDNA for detecting EC mutations, indicating that uterine-directed liquid biopsy may complement or outperform blood-based assays in certain settings (92).

Notably, several challenges currently limit clinical translation of ctDNA in EC, including the lack of standardized assay protocols, the comparatively low mutational burden and tumor heterogeneity of EC relative to other solid tumors, and interference from background normal-cell DNA; together, these factors constrain assay sensitivity and specificity (88, 93). Tumor-informed assays (e.g., Signatera) require prior knowledge of tumor-specific mutations and cannot be retrospectively applied, whereas tumor-naïve/methylation-based platforms sacrifice some sensitivity for broader applicability. Direct comparative data between these approaches in EC remain limited. In addition, no consensus threshold for MRD has yet been established for EC specifically (93). Consequently, while ctDNA has been proposed as a tool to guide escalation or de-escalation of adjuvant therapy, to date, no prospective interventional study has demonstrated that ctDNA-guided treatment de-escalation is safe or improves clinical outcomes in EC. This application therefore remains investigational and requires prospective validation before clinical implementation.

### 10.2 Single-cell RNA sequencing (scRNA-seq) and spatial transcriptomics

scRNA-seq and spatial transcriptomics have begun to transform our understanding of the EC tumor microenvironment at unprecedented resolution. Integrated scRNA-seq and spatial transcriptomics analysis in EC patients responding versus not responding to anti-PD-1 therapy revealed specific immune cell populations—CD8+ cytotoxic T cells and cell–cell communication patterns between epithelial and immune compartments—that determine sensitivity to checkpoint inhibition (94). These approaches have further revealed the co-existence of multiple tumor cell states within individual EC tumors, including states resembling normal endometrial epithelium, transit-amplifying progenitors, and fully dedifferentiated cells, alongside immune, stromal, and endothelial components whose spatial organization influences biological behavior (95).

Wu et al. performed imaging mass spectrometry for spatial ecostructural modeling of 40 EC cases, identifying CD90+ CD105+ endothelial cell populations as key predictors of tumor heterogeneity and recurrence (96). Comprehensive integration of IHC, scRNA-seq, and spatial transcriptomics has provided mechanistic insights into how the EC tumor microenvironment shapes therapeutic response and immune escape (97). These technologies are moving from exploratory research tools toward clinical applications: spatially resolved biomarker panels may soon identify the microanatomical niches in which CSCs, immune evasion, and therapy resistance are concentrated. Despite promising early results, prospective validation in larger cohorts and standardization of analytical pipelines remain necessary before scRNA-seq and spatial transcriptomics can be routinely integrated into clinical practice.

### 10.3 AI and digital pathology

AI-augmented digital pathology is rapidly entering EC diagnostics and risk stratification. HECTOR, a multimodal deep learning model introduced by Volinsky-Fremond et al. in 2024, predicts distant recurrence using routinely available H&E-stained whole-slide images and tumor stage, achieving C-indices of 0.789–0.828 across multiple validation cohorts (98). AI-based histopathology image analysis has identified a distinct aggressive subset within the NSMP (i.e., no specific molecular profile) molecular subtype, a group comprising approximately half of all diagnosed EC patients currently without clear molecular guidance, offering a cost-effective stratification approach in settings where molecular testing is unavailable (99). Deep convolutional neural networks trained to classify endometrioid endometrial carcinoma from H&E-stained slides have achieved a positive predictive value of 95.13% and an F1-score of 0.95 (100). These tools appear promising to reduce interobserver variability in EC grading, complement molecular classification, and improve accessibility of precision oncology in resource-limited settings. However, before routine clinical deployment, prospective multicenter validation, regulatory clearance, and integration into pathology workflows are required.

## 11 Integrated biological reprogramming in EC

The preceding sections address WNT/mTOR/NOTCH signaling, subcellular protein redistribution, stemness markers, redox biology, miRNA regulation, and immune reprogramming largely as parallel axes. Emerging evidence, however, indicates that these dimensions are mechanistically interconnected and that EC biology is better understood as a single, coordinated reprogramming process than as a set of isolated alterations.

Signaling convergence on differentiation and stemness. WNT/β-catenin, mTOR, and NOTCH each regulate the differentiation–dedifferentiation axis but through distinct and partly opposing logic in EC. WNT activation sustains proliferation and CSC properties (14); mTOR integrates growth factor and metabolic signals to drive proliferation and, via nuclear translocation, transcriptional reprogramming (44–46); NOTCH, in contrast, appears globally attenuated relative to normal endometrium yet paradoxically retains adverse prognostic value at higher intratumoral levels in transformed tissue (63–66). Mechanistically, the dual regulatory relationship of mTOR with WNT, with mTOR activating WNT transcriptional output in some contexts while suppressing WNT receptor levels via mTORC1/Dishevelled in others (41, 42), provides one plausible node at which these pathways may intersect to together influence proliferative and stemness phenotypes, rather than acting as fully independent oncogenic drivers.

Subcellular redistribution as functional reprogramming, not incidental relocalization. The grade-dependent compartmental shifts, including membranous-to-cytoplasmic WNT-1, cytoplasmic-to-nuclear mTOR, membranous-to-nuclear CD133, and apical-to-diffuse GGT (16–18), are not merely markers of dedifferentiation but plausibly represent gain-of-function events. Nuclear CD133, for instance, is mechanistically linked to STAT3-driven transcriptional activity in hepatocellular carcinoma, where IL-6/STAT3 signaling upregulates CD133 expression and sustains stemness under hypoxic conditions (49). Although this specific mechanism has not yet been tested directly in EC tissue, it implies that CD133 participates in an active transcriptional circuit, rather than purely functioning as a static structural marker, once relocalized to the nuclear compartment. Viewed this way, subcellular redistribution functions as a reprogramming mechanism in its own right, a parallel, protein-trafficking-based layer of regulation operating alongside transcriptional and epigenetic reprogramming.

Stemness, heterogeneity, and treatment resistance. CSC markers, including CD133, SOX2, and OCT4, intersect with the aforementioned signaling axes: WNT and mTOR activation are established drivers of stem-like states in other tumor types, where mTORC1-dependent metabolic reprogramming has been shown to sustain the self-renewal and anti-apoptotic phenotype of the stem-like compartment (47); whether this same mechanism operates in EC remains to be tested. The grade-dependent divergence of SOX2 from OCT4 co-expression (52) suggests that as EC dedifferentiates, its stem-like compartment becomes transcriptionally decoupled from canonical pluripotency circuitry.

Rather than representing a fixed, hierarchically stable subpopulation, accumulating evidence from other solid tumors indicates that CSC identity is a dynamic, environmentally and epigenetically inducible state: non-stem tumor cells can reacquire stem-like properties under metabolic, hypoxic, or therapeutic stress, and this bidirectional plasticity, rather than the mere presence of a static CSC pool, is now considered a principal driver of intratumoral heterogeneity, invasive capacity, and post-treatment recurrence (51). Applied to the EC findings above, this framework suggests that the WNT/mTOR-high, nuclear-CD133-positive, SOX2-high cell populations described in Sections 4–5 may represent an inducible state that a proportion of tumor cells can enter and exit, rather than a lineage-committed subclone—a distinction with direct implications for treatment resistance, considering that therapies targeting only the CSC phenotype at a single time point could fail if surviving non-CSC cells subsequently reacquire stem-like properties under treatment-induced selective pressure.

This is consistent with the marked intratumoral heterogeneity revealed by single-cell and spatial transcriptomic studies, which have identified coexisting tumor cell states ranging from normal-like and transit-amplifying to fully dedifferentiated phenotypes within individual EC tumors (95). Such cellular plasticity may provide a mechanistic basis for tumor invasion, recurrence, and therapeutic resistance. However, direct causal evidence linking specific alterations in cellular states or cancer stemness to treatment resistance in EC remains lacking, and the plasticity model outlined above, like the mTOR/stemness/metabolism association discussed in Section 4.2, is extrapolated from non-gynecologic tumor systems rather than established in EC-derived models.

Immune reprogramming as a downstream consequence of tumor-intrinsic signaling. The TIL and TCR data discussed earlier should not be viewed as an independent axis but as a downstream readout of the signaling state described in preceding sections. In melanoma and general T-cell biology models, WNT/β-catenin activation suppresses CCL4-dependent dendritic cell recruitment, thereby promoting T-cell exclusion (26); mTORC1 signaling shapes effector versus regulatory T-cell lineage commitment (27); and NOTCH directly regulates T-cell development and polarization, in addition to its tumor-intrinsic role. Whether these specific mechanisms operate in the EC tumor microenvironment has not been established, but if applicable, they would imply that tumor-intrinsic signaling dysregulation and immune microenvironment composition are mechanistically coupled: a tumor with high WNT/mTOR activity is possibly predisposed toward an immune-excluded phenotype through mechanisms distinct from MMR status or TMB, which are conventionally used to predict immunotherapy response in EC. This may partly explain the immunotherapy response heterogeneity observed even within molecularly defined EC subgroups (10–12).

An integrated conceptual model. Altogether, we propose that EC progression is driven by a coordinated reprogramming program in which (i) WNT/mTOR/NOTCH signaling sets the differentiation state and proliferative drive; (ii) subcellular protein trafficking may contribute to functional reprogramming by converting otherwise static expression changes into active gain-of-function signals; (iii) stemness and epigenetic (miRNA) reprogramming maintain the heterogeneous, plastic cell-state populations that this signaling supports; and (iv) these same signaling alterations shape the immune microenvironment, collectively determining both intrinsic tumor behavior and immunotherapy responsiveness. Each of these dimensions is currently assessed largely in isolation in both research and clinical practice; the multidimensional stratification framework proposed in the following section is intended as a first step toward capturing their interdependence rather than treating them as a checklist of independent biomarkers.

## 12 Clinical implications: toward multidimensional patient stratification

The molecular and immunological evidence reviewed converges on a practical, four-stage framework for clinical translation in EC, integrating tissue-based molecular classification, miRNA-augmented grade verification, ctDNA-based MRD monitoring, and biomarker-informed treatment personalization (**Figure 3**). The themes elaborated below correspond to the rationale and supporting evidence for each stage of this workflow.

**Figure 3:**
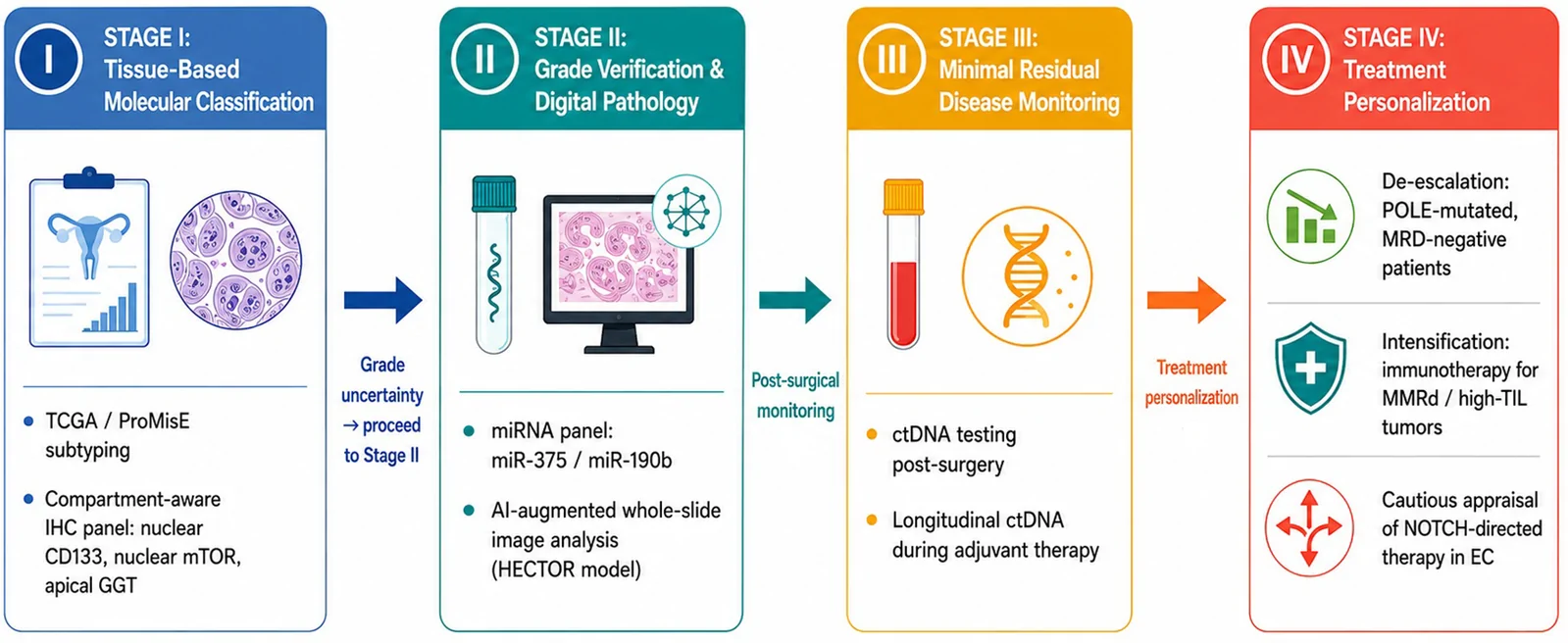
Clinical translation pathway: from molecular biomarker to precision oncology decision. Flow diagram illustrating proposed clinical integration of the reviewed molecular axes. Stage I: tissue-based molecular classification. Stage II: miRNA-based grade verification (miR-375/-190b panel) and AI-augmented whole-slide image analysis. Stage III: ctDNA-based MRD monitoring post-surgery and during adjuvant therapy. Stage IV: treatment personalization.

Subcellular protein localization as a prognostic dimension. Standard immunohistochemical analyses focused on membrane expression seem to substantially underestimate the prognostic signals embedded in protein distribution. Nuclear translocation of CD133 and mTOR, cytoplasmic redistribution of WNT-1, and loss of apical GGT each constitute grade- and stage-associated signals not captured by conventional scoring (16–18). Incorporating compartment-aware scoring protocols into routine EC pathological assessment could enhance predictive value without requiring additional assays (**Figure 2**). Although current evidence remains predominantly exploratory and derived from retrospective cohorts, compartment-specific protein localization may represent an additional layer of biologically relevant information complementary to conventional molecular classification.

Molecular grading adjuncts. miRNA profiling demonstrates that molecular signatures can provide reproducible, quantitative grade discrimination with error rates substantially lower than conventional histomorphology. A prospectively validated miRNA-based grading assay anchored on miR-375 and -190b, ideally combined with TCGA molecular classification, could reduce the interobserver variability that limits EC grading reproducibility, particularly at the G2/G3 boundary (70). The molecular heterogeneity of G2 identified by unsupervised clustering supports further stratification of this population, which currently lacks a clear clinical decision framework.

Immunotherapy biomarker refinement. Although chemo-immunotherapy has been established as a new first-line treatment paradigm, with regulatory indications differing according to MMR status (10–12), substantial variations in benefit exist across patients. TIL composition and density, WNT activation status, NOTCH pathway dysregulation, and ctDNA MRD status may collectively define composite immune-molecular signatures with superior predictive performance compared to any single biomarker. Standardized TIL scoring and ctDNA-based MRD monitoring should be integrated into prospective trial design.

NOTCH inhibition safety. NOTCH receptor expression in EC is globally reduced relative to normal endometrium, yet higher intratumoral NOTCH expression carries adverse prognostic value (63–66). Direct extrapolation of NOTCH-targeted strategies from colorectal cancer or other tumor types, where pathway behavior is more uniformly oncogenic, to EC without EC-specific mechanistic validation would be premature. The role of NOTCH inhibitors in EC requires dedicated investigation.

AI and digital pathology integration. Models such as HECTOR offer prognostic stratification from routine H&E slides, particularly relevant where molecular testing is inaccessible (98–100). Prospective multicenter validation and integration into decision support workflows alongside molecular classifiers should be prioritized.

ctDNA in clinical practice. ctDNA-based MRD monitoring may enable earlier molecular detection of recurrence and longitudinal assessment of disease burden (88–90). However, assays remain insufficiently standardized in EC, sensitivity is variable, and no prospective interventional study has established the safety or clinical benefit of ctDNA-guided treatment de-escalation. Accordingly, ctDNA should currently be regarded as an investigational biomarker for prospective clinical trials rather than a component of routine EC follow-up.

## 13 Conclusions

EC is a biologically heterogeneous malignancy whose molecular complexity extends well beyond the genomic alterations captured by current classification systems. Rather than representing a set of isolated findings, the evidence reviewed here supports a central message: EC biology cannot be comprehensively explained by genomic alterations in isolation but should be understood as a coordinated, multidimensional reprogramming process spanning signaling, subcellular, epigenetic, and immune dimensions. Aberrant developmental signaling (WNT/β-catenin, mTOR, and NOTCH) sets the differentiation state; subcellular protein redistribution may convert these signals into active gain-of-function events; miRNA dysregulation and stemness reprogramming may help maintain the resulting heterogeneous, plastic tumor cell populations; and the same signaling alterations may shape the tumor–immune microenvironment, jointly influencing intrinsic tumor behavior and immunotherapy response. Several key themes emerge: subcellular protein localization carries independent prognostic information that is not captured by conventional membrane-focused immunohistochemical scoring. The expanding tissue and circulating miRNA literature, of which our own G2/G3 panel is one contribution among many (21, 22), offers an increasingly validated adjunct to histomorphological grading. NOTCH receptor expression in EC shows global reduction relative to normal endometrium combined with adverse prognostic value at higher intratumoral levels, a context-dependent pattern that complicates direct translation of NOTCH-targeted strategies from other tumor types (67); the tumor–immune microenvironment, probably shaped by the same signaling pathways driving intrinsic tumor reprogramming, is now a therapeutically targeted compartment, with three landmark approvals transforming the first-line treatment paradigm for advanced disease, albeit with regulatory indications that differ by MMR status. Finally, ctDNA-based MRD monitoring, spatial transcriptomics, TCR repertoire profiling, and AI-augmented digital pathology represent a rapidly maturing but still largely exploratory and retrospective frontier. Realizing the clinical potential of this integrated framework will require validation in prospective, multicenter cohorts, functional studies that establish causal rather than merely correlative relationships among these molecular dimensions, and sustained multidisciplinary research efforts.
